# Smart material based on boron crosslinked polymers with potential applications in cancer radiation therapy

**DOI:** 10.1038/s41598-021-91413-x

**Published:** 2021-06-10

**Authors:** José Vedelago, Facundo Mattea, Sebastián Triviño, María del Mar Montesinos, Walter Keil, Mauro Valente, Marcelo Romero

**Affiliations:** 1 Instituto de Física Enrique Gaviola (IFEG), CONICET, Córdoba, X5000HUA Argentina; 2 Laboratorio de Investigación e Instrumentación en Física Aplicada a la Medicina e Imágenes por Rayos X (LIIFAMIRx), FAMAF-UNC, Córdoba, X5000HUA Argentina; 3 Departamento de Química Orgánica, FCQ-UNC, Córdoba, X5000HUA Argentina; 4 Instituto de Investigación y Desarrollo en Ingeniería de Procesos y Química Aplicada (IPQA), CONICET, Córdoba, X5000HUA Argentina; 5 Centro de Medicina Nuclear y Radioterapia Patagonia Austral (CEMNPA), Río Gallegos, Z9400 Argentina; 6 FCEFyN-UNC & CNEA-Reactor Nuclear RA-0, Córdoba, X5000HUA Argentina; 7 Departamento de Bioquímica Clínica, Centro de Investigaciones en Bioquímica Clínica e Inmunología (CIBICI), CONICET, FCQ-UNC, Córdoba, X5000HUA Argentina; 8 Departamento de Ciencias Físicas, Centro de Física e Ingeniería en Medicina (CFIM), Universidad de La Frontera, Casilla 54-D, Temuco, Chile; 9 Present Address: Division of Medical Physics in Radiation Oncology, German Cancer Research Center (DKFZ), 69120 Heidelberg, Germany

**Keywords:** Actuators, Sensors and biosensors

## Abstract

Organoboron compounds have been playing an increasingly important role in analytical chemistry, material science, health applications, and particularly as functional polymers like boron carriers for cancer therapy. There are two main applications of boron isotopes in radiation cancer therapy, Boron Neutron Capture Therapy and Proton Boron Fusion Therapy. In this study, a novel and original material consisting of a three-dimensional polymer network crosslinked with $^{10}$B enriched boric acid molecules is proposed and synthesized. The effects of the exposition to thermal neutrons were studied analyzing changes in the mechanical properties of the proposed material. Dedicated Monte Carlo simulations, based on MCNP and FLUKA main codes, were performed to characterize interactions of the proposed material with neutrons, photons, and charged particles typically present in mixed fields in nuclear reactor irradiations. Experimental results and Monte Carlo simulations were in agreement, thus justifying further studies of this promising material.

## Introduction

New trends in oncological radiation therapy are centered on the use of particles like neutrons or protons, rather than using high energy photon beams, mainly because of their precise energy deposition and minimum damage to healthy tissue^1,2^. These novel treatments require a proper quality assurance to verify the prescribed treatment on patients and also the design of new biocompatible materials that serve as carriers of markers, contrast or treatment enhancers^3,4^. Polymers represent one of the most suitable and versatile option for that purpose, together with nanocomposites, nanoparticle systems, and lipid nanoassemblies^5–9^. Due to the large number of possible modification reactions and their similar atomic composition to human tissue, they can be designed and used in combination with different agents with a specific application in mind.

Polymers based on 2-Hydroxyethyl methacrylate (HEMA) have been used in several biological applications ranging from drug delivery to the design of functional contact lenses^10,11^. HEMA biocompatibility has been exploited in biological systems to design functional materials with smart responses to different stimuli^12,13^. Furthermore, organoboron compounds have been playing an increasingly important role in analytical chemistry, organic synthesis and catalysis, drug delivery, material science, optoelectronic applications, and functional polymers used as boron carriers for cancer therapy^14–16^.

There are two main potential applications of boron isotopes in radiation cancer therapy, the well established Boron Neutron Capture Therapy (BNCT)^17^ and the conceptual application of Proton Boron Fusion Therapy (PBFT), which has recently been experimentally proved by Cirrone et al.^18^ and theoretically explored by Geser and Valente^19^. On one hand, BNCT treatment has the capacity of delivering high dose levels in the proximity of boron atoms due to the high thermal neutron interaction cross section of $^{10}$B^20^. As a consequence of this nuclear reaction, an alpha particle and a $^7$Li ion along with a 478 keV photon are emitted in 94 % of the cases. In the remaining 6 % of the cases, also an alpha particle and a $^7$Li ion are produced. These high linear energy transfer particles have enough energy to interact with atoms within a range of ten micrometers which is less than the size of most human cells^21^. Therefore, in BNCT treatments, only cells doped with $^{10}$B become affected. Additionally, the 478 keV prompt gamma emission offers the possibility of external monitoring of the delivered dose^22,23^. On the other hand, in PBFT three alpha particles with a 30 $\upmu$m range are generated thanks to the high interaction cross section of $^{11}$B with protons^18,24^. Additionally, real-time monitoring of the treatment can be performed thanks to a 719 keV prompt gamma emitted in the proton boron fusion reaction^25^.

In PBFT and BNCT applications is essential to locate the boron compound in the proximity or within the tumor tissue, and to achieve high in-tumor boron concentration stability during oncological treatments^17,18^. There are two main potential ways of introducing the material inside patients. One of them is to modify the material in order to link targeting agents to the polymer molecule, then the polymer would be selectively distributed among tumor tissue, as has been already done in other techniques^8,26–28^. On the other hand, the material could be introduced in the patient as a liquid suspension, within the tumor tissue with the assistance of imaging methods, as proposed for other oncological treatments, such as endoscopic ultrasound-guided fine-needle aspiration^29,30^, or by intratumoral injections of hydrogels to achieve the sustained release of a drug^31^.

Neutron sources, such as nuclear fission reactors, accelerators and radioactive sources, mainly produce a mixture of gamma rays and neutrons with a broad energy spectrum^32^. Moderation filters are typically used to increase the relative amount of thermal and epithermal neutrons by decreasing the kinetic energy of fast neutrons. However, the filtering itself increases the gamma radiation background that reaches to the target, which is therefore exposed to a mixed field. Thus, a suitable detection and dosimetry system is necessary to discriminate each particle contribution in the total absorbed energy.

Most current neutron detectors are able to measure neutron fluence and then calculate each component dose contribution. Typical examples of these detectors are activation foils^33^ or fluorescent nuclear track detectors (FNTD)^34^. Another type of dosimetry system which has been widely used in neutron detection is thermoluminescent dosimetry, such as LiF (TLD-100), $^7$LiF (TLD-700) and $^6$LiF (TLD-600)^35^, which require the subtraction of the gamma contribution to estimate the neutron dose. What is more, there are few reports on thermoluminescent materials based on synthetic polycrystals of CaSiO$_3$^36^, which are able to provide a direct measurement of neutron fluence. In the last two years, scintillation detectors have been proposed for the discrimination of neutrons in mixed fields by means of adequate shielding and signal post-processing^37^. In medical physics, gel dosimeters doped with compounds based on $^{10}$B are used to mimic BNCT treatments and thus quantifying the neutron capture dose contribution^38^. Although many of these detection systems are capable of determining the neutron contribution, most of them still require signal post-processing and calculations in order to discriminate the neutron contribution to the total absorbed dose^39,40^.

In this study, a novel and original material consisting of a three-dimensional polymer network crosslinked with molecules containing $^{10}$B atoms is proposed and synthesized. Then, the effects of exposing this material to thermal neutrons were studied. Also, the potential capabilities of the developed material as sensitive material for neutron detection and as a controlled boron delivery system were preliminary studied. The proposed material is intended for BNCT applications as a radiosensitive material for thermal neutrons and/or as an alternative to current drug delivery systems. It could also be applied to protontherapy as an alternative boron-based compound capable of online monitoring along with local dose enhancement due to proton–boron fusion. Moreover, this material may represent an alternative for boron-based drug delivery in PBFT. Once the proton beam thermalizes around the Bragg peak, usually within the target volume, the $^{11}$B content is useful for proton-boron fusion, thus providing both an extra high LET local dose enhancement and a chance for online monitoring by means of the 719 keV prompt gamma emissions.

The hypothesis of the present study is that alterations in the molecular structure of the material would be generated as a consequence of the neutron capture reactions, then these modifications would produce changes in the mechanical properties of the macroscopic material. In this way, the number of neutrons being captured by $^{10}$B should be proportional to the decrease of the crosslinking degree, and consequently to the decrease in the elastic modulus of the material. The main goal of this investigation is to design, synthesize and characterize a new material sensitive to thermal neutrons while maintaining a similar atomic composition to the typical tissues involved in BNCT.

## Results and discussion

### Poly(HEMA)$^{10}$B synthesis

**Figure 1:**
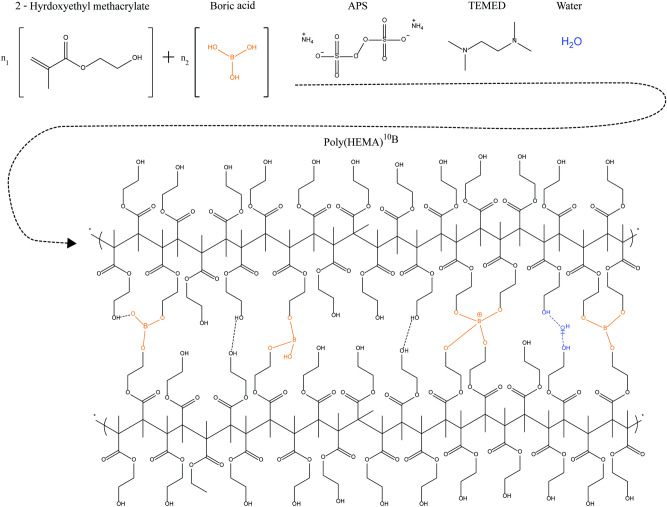
Reaction scheme of the new material.

**Figure 2:**
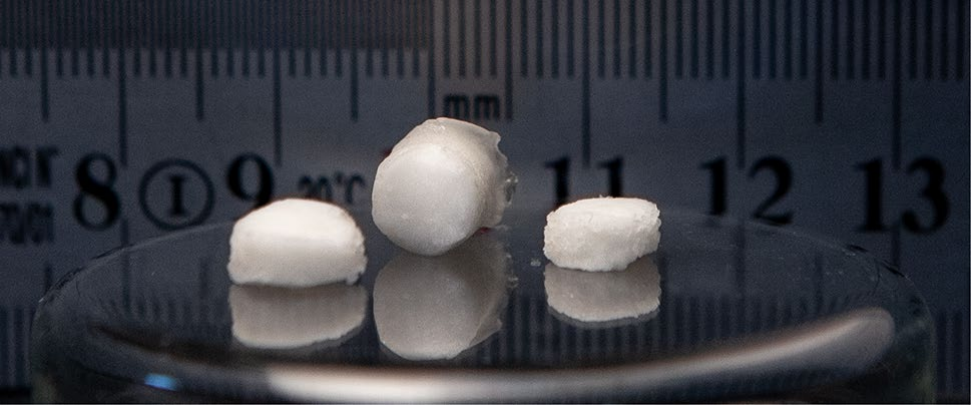
Poly(HEMA)$^{10}$B samples.

**Figure 3:**
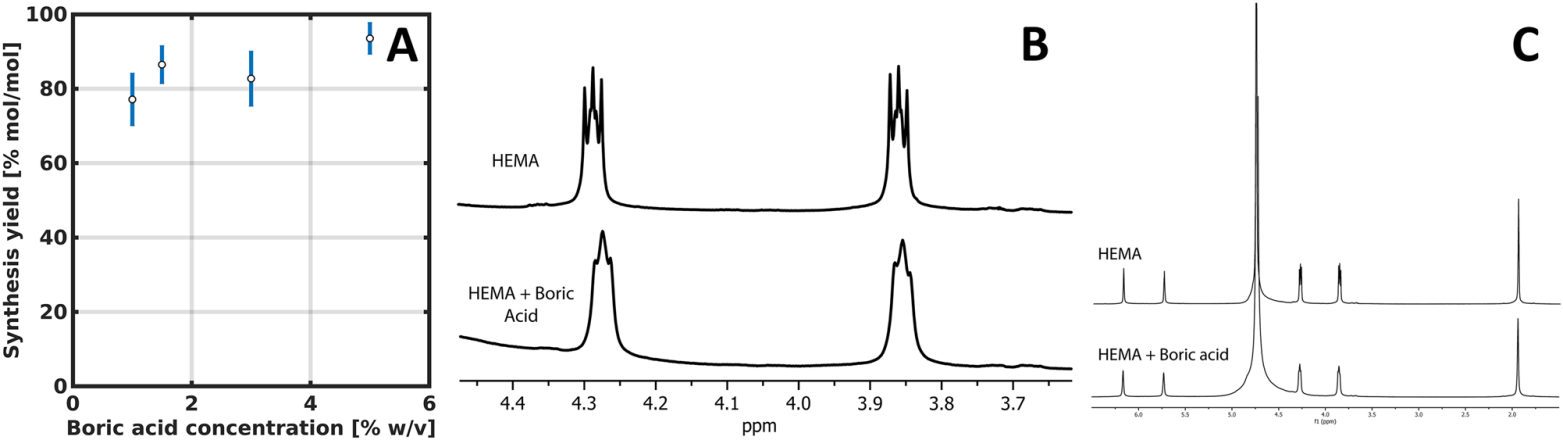
Synthesis yields for different concentrations of boric acid **(A)** and NMR analysis of the interaction between HEMA and boric acid **(B,C)**.

A new polymeric material, named poly(HEMA)$^{10}$B, was synthesized from the acrylic monomer HEMA using boric acid-$^{10}$B with 99 atom % of $^{10}$B as a crosslinking agent. The proposed reaction scheme is depicted in Fig. 1. In all syntheses, elastic solid materials with a rubbery appearance were obtained as shown in Fig. 2. The synthesis products consisted on a sponge-like solid and a liquid phase, it is well known that the structure of poly(HEMA) networks is highly dependent on the water content of the polymerization mixture, and although monomeric HEMA is soluble in water, poly(HEMA) is not and has limited compatibility with the solvent^41,42^. The synthesis yields in the poly(HEMA)$^{10}$B synthesis with different concentrations of boric acid, defined as the amount of HEMA included in the polymeric material over the total amount of HEMA in the synthesis, are depicted in Fig. 3A. A higher boric acid concentration leads to higher reaction yields, and the highest achieved reaction yield resulted in more than 93 % mol/mol.

In this work we propose a different method than the ones usually reported in bibliography, in which hydroxylated polymers like polyvinyl alcohol (PVA) reacts with boron forming networks with a low crosslinking degree and weak mechanical properties^43^ as a result of conformational restrictions in the macromolecule. Conversely, the reaction mechanism of the proposed synthesis involves the formation of a borane ester between the OH-group of the monomer HEMA and boric acid. During the elongation of the poly(HEMA) chain, the interchain borane junctions are formed by didiol complexes with boron and the simultaneous crosslinking during the radical polymerization. With this methodology, the obtained products showed improved mechanical properties in comparison with materials based on direct polymeric reactions^43^. Moreover, the initial interaction between HEMA and boric acid before the polymerization reaction was studied by nuclear magnetic resonance (NMR). As depicted in the spectrum of Fig. 3C no new signals or relevant changes in the integration or shifts were found that could indicate alterations in the protons of the HEMA, discarding any other possible reaction than the proposed one. However, the HEMA proton expected to interact with boron belongs to the OH-group, which is mobile and cannot be detected because it is interchangeable with deuterium atoms of the solvent. Nonetheless, when the spectrum is magnified (Fig. 3B) the addition of boron to the HEMA molecule can be observed as a broadening in the proton signals of HEMA. For example, a similar broadening in the NMR analysis of the reaction between boric acid and hyaluronic acid has been reported by Zelenetskii et al.^44^ and attributed to the formation of a complex between the two molecules. What is more, the multiplicity changes in the NMR spectra indicate a conformational restriction of the carbon chain of HEMA, which after interacting with boron, became restrained from rotating.

**Figure 4:**
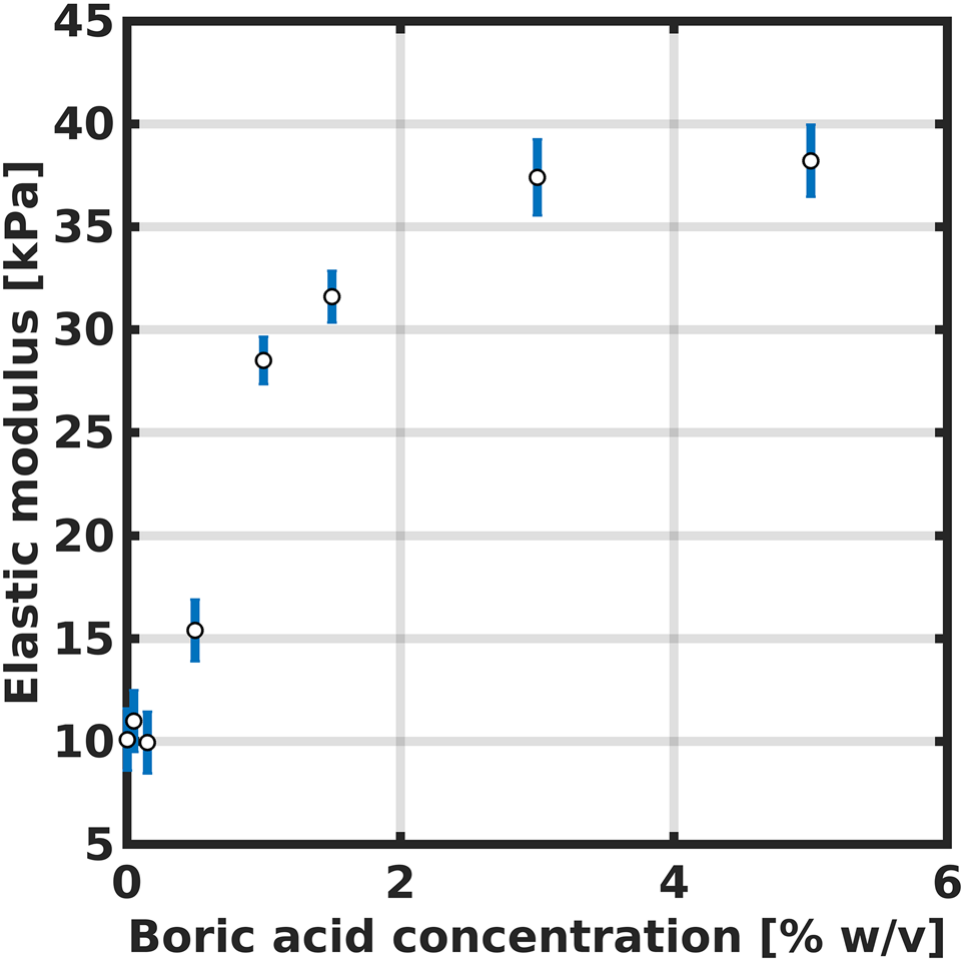
Elastic modulus for the synthesized poly(HEMA)$^{10}$B.

The rheological properties of the poly(HEMA)$^{10}$B followed the same trend than the reaction yields. As reported in Fig. 4, a higher concentration of boric acid leads to a higher elastic modulus of the crosslinked material, reaching a saturation behaviour for concentrations near 5 % w/v, with an elastic modulus of 38 kPa. Also, a minimum concentration of 0.5 % w/v was needed in order to obtain a crosslinked viscoelastic material. This behavior is commonly observed in polymeric viscoelastic materials and has been reported by several authors^45,46^.

These results indicate that boric acid is acting as a crosslinking agent in the HEMA polymeric network, also validated by the interaction between the monomers in the NMR spectroscopy results.

### Poly(HEMA)$^{10}$B as controlled boron delivery system

**Figure 5:**
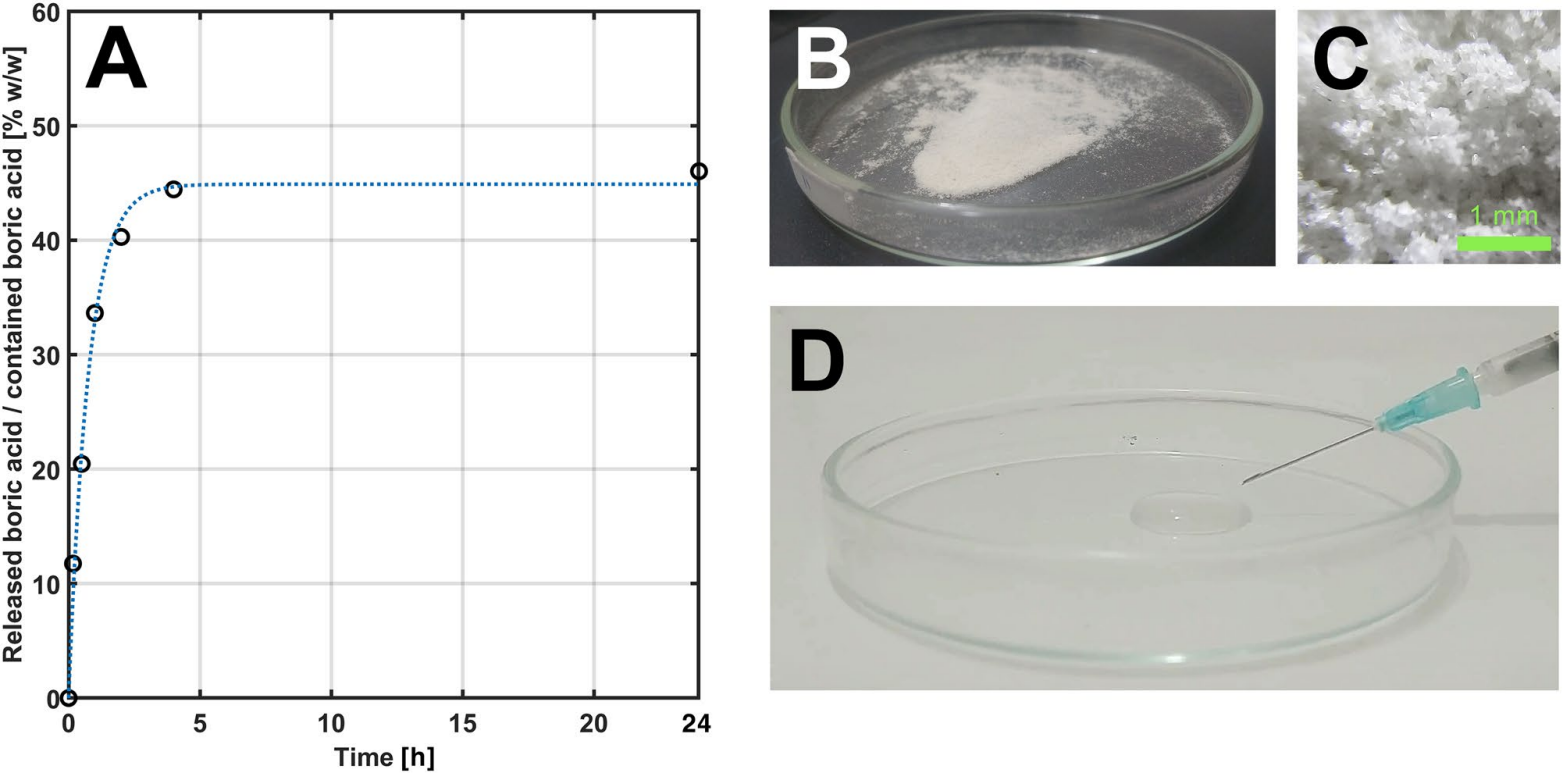
**(A)** Boric acid release curve, experimental data (o) and fitted a pseudo-first-order kinetics empiric model (dotted line). **(B)** Powder of poly(HEMA)$^{10}$B material with particle size below 150 $\upmu$m. (**C**) Particle morphology as obtained from micro-photography. **(D)** Flowing test in a 21-G needle of an SBF suspension of the poly(HEMA)$^{10}$B particles.

The release kinetics of boron delivery due to the degradation of poly(HEMA)$^{10}$B in contact with simulated body fluid (SBF)^47^ and maintained at typical body temperature (37 $^\circ$C), was investigated. Fig. 5A depicts the delivery of boron at different times and a pseudo-first-order kinetics model^48^ was used to fit the experimental results. The delivery mechanism that estimates the delivery of boron with a reasonable agreement with the experimental data is known as controlled delivery by bulk network degradation^49^. The kinetic constant was estimated, with a confidence of 95 %, as 1.323 h$^{-1}$. Therefore, the mean lifetime corresponding to 50 % of the (asymptotic) maximum boron delivery is 0.52 h. According to other authors, these parameters are useful to describe the degradation of polymeric materials by hydrolysis of many polyesters, like polyethylene glycol, polylactic acid and poly lactic-co-glycolic acid^50^, and the degradation mechanism known as bulk erosion degradation has been widely exploited for controlled drug delivery^51^.

It is worthwhile mentioning that, after the delivery of boron, the expected material in aqueous solution should be just HEMA homopolymer and boric acid. Actually, this polymer is broadly used as the main component of contact lenses as well as to improve cell adhesion^52^, both proofs of the biocompatibility of the polymer. Furthermore, polyHEMA metabolism was studied using gingival fibroblast cells^53^ obtaining primary degradation molecules of methacrylic acid and ethylene glycol, and then formaldehyde and pyruvate as final products. In addition, in this process only a weak cell viability diminution was observed, indicating the low cytotoxicity of polyHEMA, indicating that the resulting material after drug delivery of boron should also be biocompatible.

Parenteral administration using a needle and a syringe or by catheter insertion are typically used as possible routes of similar hydrogel and polymeric materials. In this framework, the dehydrated poly(HEMA)$^{10}$B material was micronized, resulting in particle size below 150 $\upmu$m (Fig. 5B,C), suspended in SBF solution and finally passed through a standard needle (Fig. 5D). (A video of the flow test is included as supplementary information online).

Thereby, the capability of micronizing and preparing poly(HEMA)$^{10}$B suspension enables the use of targeting and delivery techniques like image-guided methods, which could efficiently locate the needle in the target site. Several examples of similar methods have been proposed like endoscopic ultrasound eco-guided fine-needle aspiration^29^, or the eco-guided intratumoral injection of a hydrogel-based on hyaluronic acid, ethylene glycol and adipic acid dihydrazide to achieve the sustained release of carboplatin improving the malignant glioma treatment^31^. In practical terms, after a careful cytotoxicity characterization, the proposed material could be considered as an alternative drug delivery system in BNCT or PBFT by injecting formulations of the synthesized material enriched with $^{10}$B or boron in natural abundance, respectively. Once an appropriate uptake is verified within the target volume, the subsequent neutron or proton irradiation could be performed. However, exhaustive in-vitro and in-vivo studies of the cytotoxic and antineoplastic properties of poly(HEMA)$^{10}$B are mandatory prior to implementing it as an alternative therapeutic drug.

### Polymer radiation response

Materials with a concentration of 5 % w/v of boric acid were used to study the radiation effects on poly(HEMA)$^{10}$B because they maximize the possible thermal neutron captures in $^{10}$B isotope. This concentration is not only near the solubility limit, but also the results of the elastic modulus of materials obtained with different boric acid concentrations indicate that more than 5 % w/v of boric acid can not be incorporated into the polymeric network.

**Figure 6:**
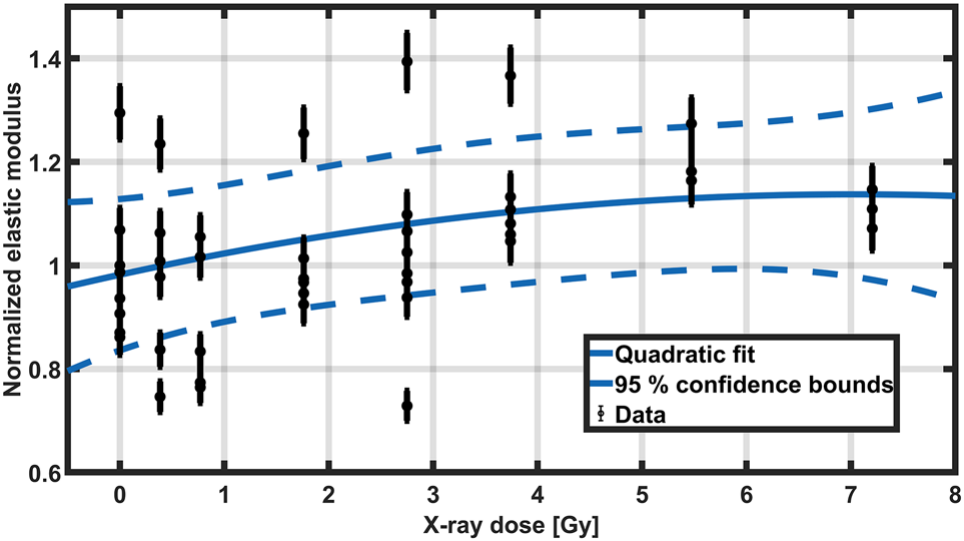
Response of poly(HEMA)$^{10}$B to X-ray beams.

The response of the material to X-rays was studied by measuring the elastic modulus of samples of poly(HEMA)$^{10}$B irradiated at different doses, depicted in Fig. 6. The results suggest that there is an influence of X-rays over the mechanical properties of the materials, increasing the elastic modulus of the poly(HEMA)$^{10}$B. However, this behaviour is not pronounced and reaches a maximum increase of 10 % for doses higher than 5.5 Gy.

Two possible phenomena could take place in the material that produce an increase in the elastic modulus. First, the unreacted HEMA during the material synthesis can undergo a radical polymerization induced by water radiolysis^54^, thus increasing the molecular weight and complexity of the crosslinked material and therefore obtaining a more rigid material. Also, other authors proved that during the homopolymerization of HEMA, crosslinked materials can be obtained if traces or impurities of a crosslinking molecule are present^42^, hence when exposed to X-ray beams higher crosslinking degrees could be obtained.

**Figure 7:**
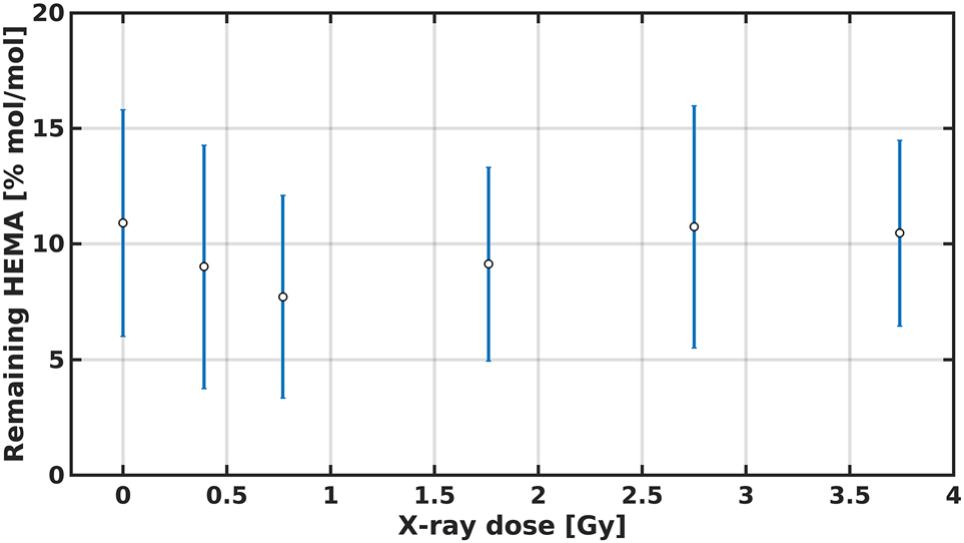
Remaining HEMA for poly(HEMA)$^{10}$B samples irradiated at different X-ray doses.

The remaining HEMA in the irradiated samples indicates that no significant amount of HEMA is being incorporated into the material during the irradiation, as shown in Fig. 7. These results suggest that the observed increase in the elastic modulus is related to the formation of a more complex material with a higher crosslinking degree.

**Figure 8:**
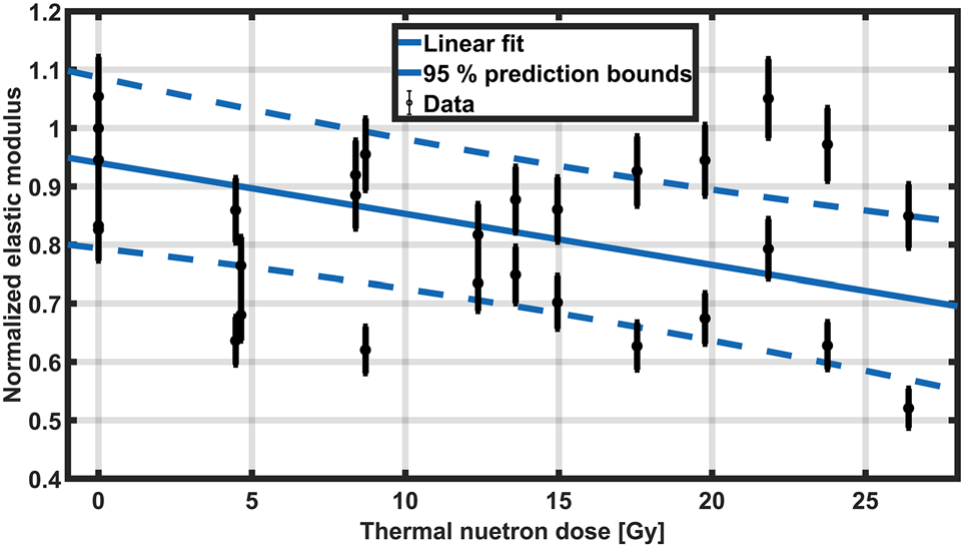
Relative elastic modulus for different contributions to the total absorbed dose due to neutron capture in $^{10}$B isotope.

Because of the obtained results, poly(HEMA)$^{10}$B samples were initially irradiated with 5.5 Gy using an X-ray tube and then exposed to mixed neutron-gamma fields. Results of the material response at different thermal neutron doses are depicted in Fig. 8. Although there is a relatively large dispersion in the response, which could be attributed to the nature of the material and to the precision of the analytical technique, the average relative elastic modulus decreases following a linear trend with an increase in the thermal neutron dose, up to a 30 % reduction within the studied range. These results agree with the proposed hypothesis, since the elastic modulus of the material decreases when a higher number of boron atoms are eliminated from the crosslinked poly(HEMA)$^{10}$B network. The analytic technique used for measuring the changes in the macroscopic mechanical properties needs to be further improved in order to reduce data dispersion.

**Figure 9:**
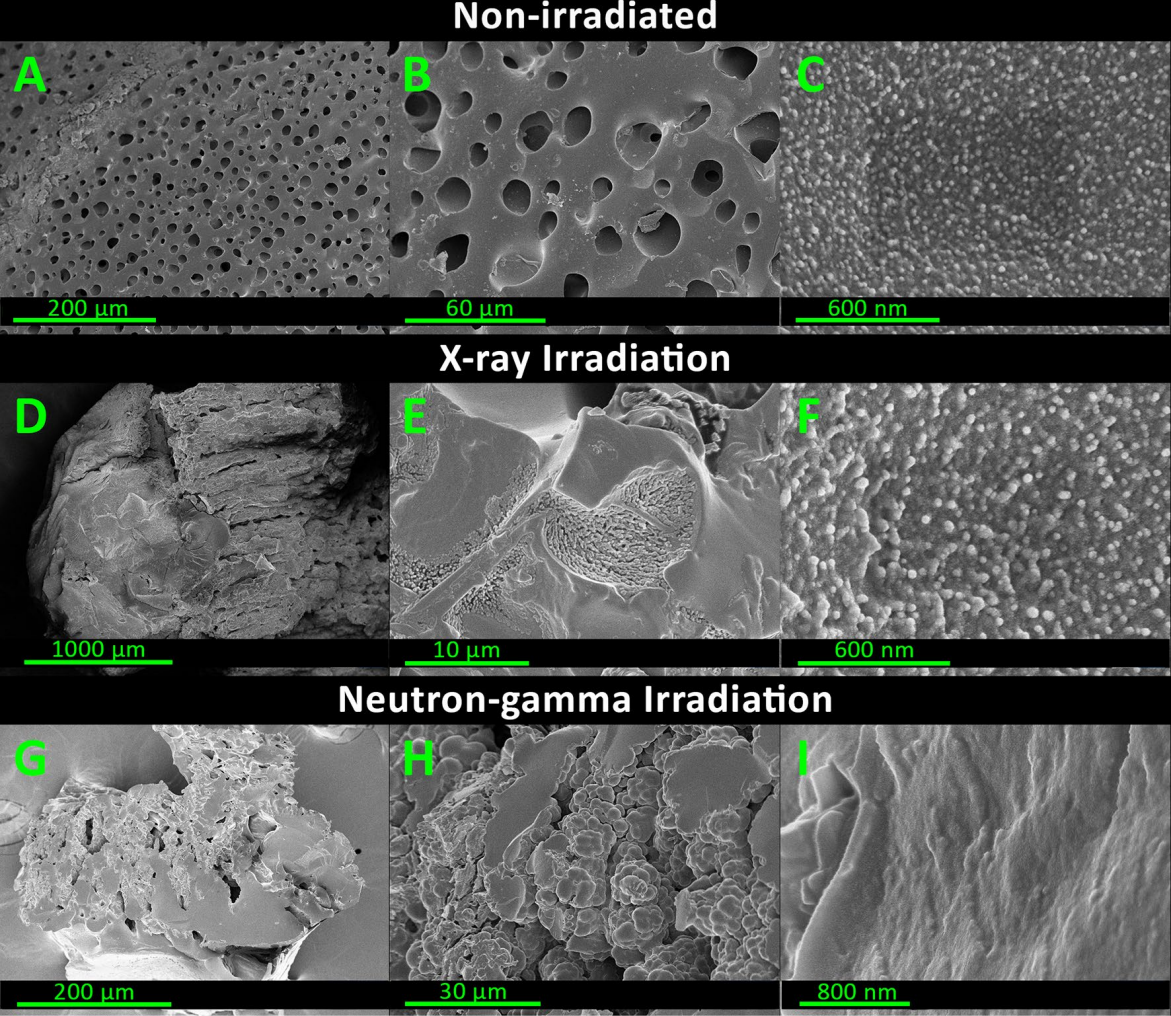
Morphological characterization of poly(HEMA)$^{10}$B samples irradiated at different conditions.

In order to verify if the variation in the elastic modulus is related to changes in the structure of the material, a morphological characterization was carried out. Images of a non-irradiated sample, a sample irradiated with X-rays and a sample irradiated with a neutron-gamma field are shown in Fig. 9. At low magnification (image A), poly(HEMA)$^{10}$B presents high porosity with an average pore diameter of 10 $\upmu$m. Pores are separated by walls of significant thickness, so that the percentage of the surface covered by pores is approximately 20 % of the total area in the images. Globular structures are observed on the surface of the wall material that could be associated with polymeric coils (image C). These structures represent the primary macromolecular arrangements used to build the whole macroscopic network. Such polymer coils are formed during the radical propagation reaction in which phase separation occurs during rapid chain growth^55^. Subsequently, these spherical organizations are responsible for the formation of the macroscopic porous structure of the material. Although samples analyzed with an intermediate magnification seem to have differences in their structure, as can be observed between images B and E, both materials still have the same conformational structure in the nanoscale as inferred from images C and F, but with larger globular cluster sizes.

Most significant differences in the material are observed in the samples exposed to the gamma-neutron field, where morphological alterations can be observed in all the analyzed samples and magnifications with respect to the non-irradiated ones. In detail, at low magnification (image G) the structure of the material is similar to those obtained after the X-ray irradiation. However, there are no polymer coils in the surface of the obtained material (image I), which becomes smooth and homogenous in contrast with the original and X-ray irradiated samples (images C and F).

A possible interpretation of these differences could be attributed to the capture of thermal neutrons with the $^{10}$B, acting as a crosslinking agent in the macromolecular structure, therefore reducing the number of crosslinking points and enhancing the conformational freedom and mobility of the polyHEMA chains. Then, after the irradiation, the material has the ability to rearrange its molecular structure and adopt a more relaxed conformation. This interpretation agrees and complements the observed elastic modulus results previously presented. After neutron irradiation, there is a decrease in the concentration of boron in the polymeric network and structurally the fall in the crosslinker concentration, which is manifested as a rearrangement and approach of macromolecular chains^56^ that is observed as the loss of globular structures, leading to a smooth surface and a more homogeneous material.

Detailed neutron transport interaction simulations using Monte Carlo codes can be used to assess the total number of $^{10}$B atoms that have undergone neutron capture, thus estimating the number of crosslinking points in the poly(HEMA)$^{10}$B network that has been destroyed, as well as the quantity of the heavy ions $^7$Li and alpha ($^4$He) particles created.

Since neutron capture in $^{10}$B produces high energy transfer alpha particles and lithium nucleus, the microscopic volume around the location of the $^{10}$B nucleus undergoing the reaction can be affected. Therefore, dedicated simulations in FLUKA Monte Carlo code were carried out to estimate the corresponding “ionization volume” following de-excitation.

**Figure 10:**
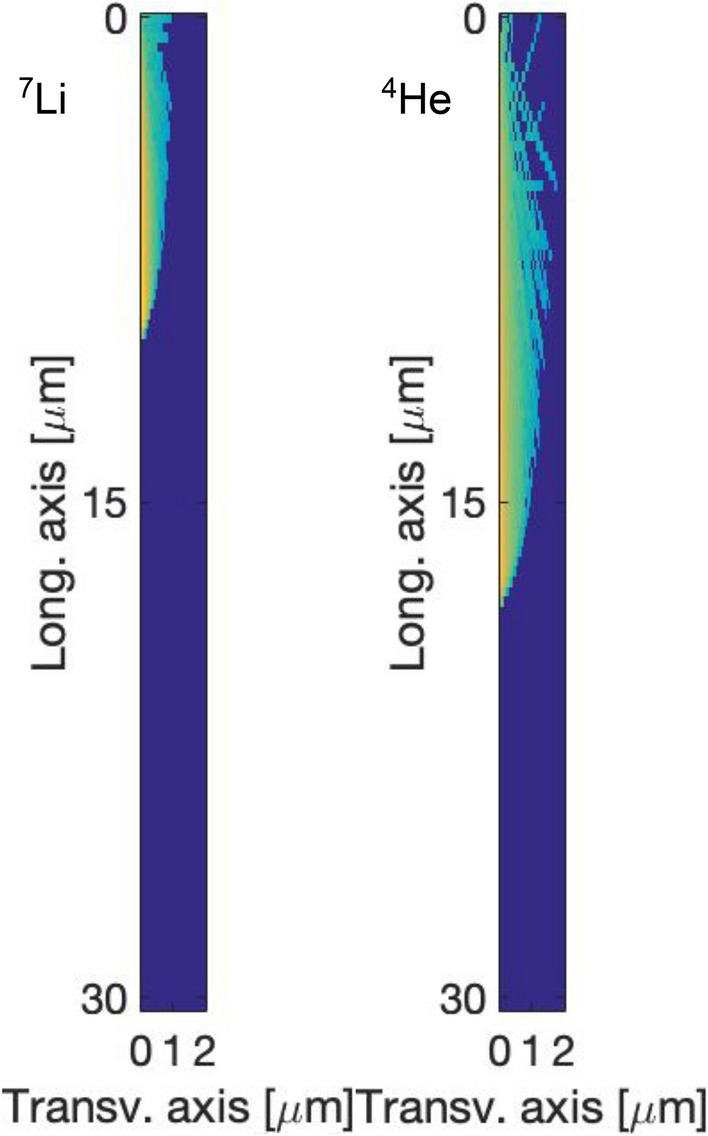
Ionization density distribution resulting from the high linear energy particles generated in boron neutron capture reactions in the poly(HEMA)$^{10}$B material.

Once confirmed that the morphological characterization supports the observed variations in the elastic modulus, it remains necessary to address why the elastic modulus decreases when samples are irradiated with neutrons. Results obtained for some physical properties of particles produced in the neutron capture interaction are reported in Fig. 10 and Table 1.

**Table 1:** Results from FLUKA simulations used for the affected volume estimation. Overall uncertainties are less than 5 % in all cases.

Particle	Range ($\upmu$m)	Lateral extension ($\upmu$m)
$^7$Li	10.2	0.91
$^4$He	17.8	0.67

In the surrounding regions of points where nuclear reactions occur, a high number of ionizations per atom or molecule are produced. According to the values reported in Table 1, the calculated percentage of affected volume due to neutron irradiation is around 33 %, considering 7.88 $\times$ 10$^{10}$ captures reactions per mL for a thermal neutron fluence of 1.55 $\times$ 10$^7$ neutrons/(cm$^2$s), as obtained by MCNP simulation.

These results suggest that neutron captures are not only eliminating the crosslinking points of the molecular structure of poly(HEMA)$^{10}$B, but also affecting up to 33 % of the material, therefore being able to produce changes in the microscopic scale. The morphological characterization of the irradiated samples agrees with this hypothesis, where the polymeric coils were only modified when irradiated in a neutron-gamma field.

**Figure 11:**
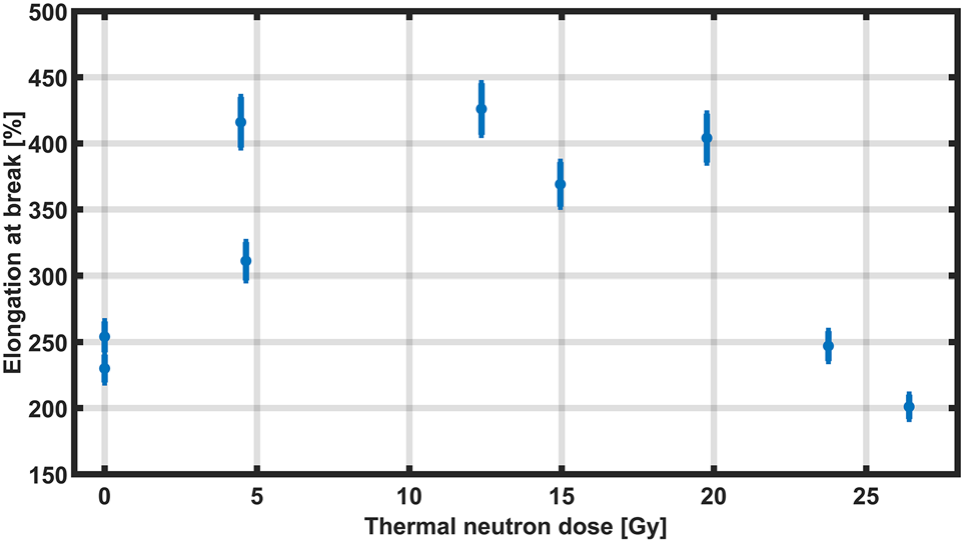
Percentage elongation at break from the tensile test of poly(HEMA)$^{10}$B.

In order to complement the microscopic characterizations and rheological studies, a tensile test was performed for samples irradiated at different neutron dose values. The obtained results are depicted in Fig. 11. The percentage of elongation observed in materials containing HEMA-boron of up to 400 % had also been described by Takeno et al.^57^ in similar materials prepared from PVA (polyvinyl alcohol)-boron. These polymers also have borane ester bonds, and the authors attributed the high elongation capacity of the material to the presence of boron. The percentage of elongation at break of the HEMA-boron samples shows an initial increase followed by a subsequent decrease (Fig. 11). Such growth and decrease behaviour was also reported by Kudoh et al.^58^ after irradiating polytetrafluoroethylene samples with gamma rays. Although this effect was observed at a higher gamma-ray dose value than the ones used in this study, the chemical composition of the polymer is different and the elongation results could also integrate the microscopic alterations caused by the neutron capture of the boron atoms present in poly(HEMA)$^{10}$B, as observed by the morphological characterization reported in Fig. 9. Similar tensile behaviour was also observed by Dubey et al.^59^ when irradiating samples of a crosslinked polymer based on a reinforced epoxy resin with gamma rays. The reported initial growth in elasticity was interpreted as a weakening of the bonds between the matrix chains macroscopically manifested as an increase in elongation. Nevertheless, the decrease in the elongation capacity of the material beyond a certain limit suggested an increase in fragility due to the formation of a rigid network^59^.

In the poly(HEMA)$^{10}$B samples, the carbon-based network is covalently crosslinked by ester-borane bonds which, when irradiated with thermal neutrons and gamma rays, produce capture events and further ionizations. The reactions produced would be responsible for the decrease in the number of ester bonds in the macromolecular chains, favouring their mobility and therefore increasing the percentage of elongation at break, as reported in Fig. 11. As the thermal neutron dose increases, a similar behaviour to that mentioned in the literature for gamma-only irradiations at doses above kGy was observed^58,59^. As discussed within the FLUKA simulations results, the high linear energy transfer particles generated in the neutron capture might be responsible for the subsequent generation of radicals, covalent crosslinking and causing the material to be more rigid, brittle and with lower elongation at break. Although the results obtained by implementing the poly(HEMA)$^{10}$B mechanical properties as a dosimetric response preliminarily suggested a correlation, the data dispersion can be further improved by increasing the total delivered dose to the samples, whereas the spatial resolution could be improved by analyzing the dynamic material deformation with atomic force microscopy, also known as microrheology mapping^60^. Since the material can be synthetized with different isotopic boron compositions, its potential application in either BNCT or PBFT becomes available, because chemical interactions do not depend on nuclear properties, thus providing a simple way of increasing the $^{10}$B or $^{11}$B relative concentration in the polymeric material. Local $^{10}$B accumulation within target volumes represents a key issue for many therapeutic applications, like BNCT and PBFT, thus it may be envisaged an implementation of poly(HEMA)$^{10}$B as an alternative for the currently used boron compounds by means of guided localized injections within the target volumes.

## Conclusions

A novel material, called poly(HEMA)$^{10}$B, has been proposed and synthesized by crosslinking 2-Hydroxyethyl methacrylate with boric acid, and its molecular structure and conformation were spectroscopically verified. The poly(HEMA)$^{10}$B material was synthesized with boric acid-enriched 99 % in the isotope $^{10}$B, obtaining a material with a concentration of 5 % w/v sensitive to thermal neutrons. Due to the $^{10}$B concentration in the enriched poly(HEMA)$^{10}$B the material exhibited structural and mechanical changes attributable to the thermal/epithermal neutron irradiation, representing a starting point for considering the poly(HEMA)$^{10}$B as a suitable radiosensitive material potentially useful for thermal/epithermal neutron detection. Reported results regarding the boron release in SBF as a product of the poly(HEMA)$^{10}$B bulk erosion are the starting point for further studies about boron delivery for radiation therapy. Although the implemented analytic technique used for measuring macroscopic mechanical properties needs to be further improved, the variations in the elastic modulus combined with the observed morphological changes support the potentiality of poly(HEMA)$^{10}$B as a selective detector in mixed field irradiations. Monte Carlo simulations of radiation transport were used to verify the extension of the neutron capture effect, proving that up to 33 % of the volume was affected during the irradiation, thus characterizing the substantial modifications produced on the morphology of the material at the micro-scale. In summary, the poly(HEMA)$^{10}$B synthesis method with boric acid acting as a crosslinking agent was reported and the response of this new material to ionizing radiation was supported by Monte Carlo simulations. According to the reported results, the development of poly(HEMA)B as a novel and potentially efficient boron delivery agent may constitute a breakthrough in order to make progress with BNCT and PBFT. Nevertheless, the pathway for its implementation still requires exhaustive studies related to its cytotoxicity and biocompatibility, the characterization of the poly(HEMA)B uptake and biodistribution in tumor cell models, as well as cell survival following in-vitro and in-vivo irradiations.

## Methods

### Poly(HEMA)$^{10}$B polymer synthesis

Acrylic monomer 2-Hydroxyethyl methacrylate (HEMA) (95 % purity) obtained from FLUKA (Honeywell International Inc.) was used in the material synthesis. Ammonium persulfate (APS) (Anedra, Argentina) and *N,N,N’,N’*-tetramethylethylenediamine (TEMED) (Sigma-Aldrich, USA) were used as initiators. Boric acid H$_3$ $^{10}$BO$_3$ containing 99 atom % $^{10}$B (Sigma-Aldrich, USA) was used as a crosslinking agent. The polymers were synthesized by means of a radical reaction from a solution containing 0.4 mL of HEMA, 20.0 mg of APS, 0.2 mL of an aqueous solution containing 0.32 M of TEMED and different amounts of H$_3$ $^{10}$BO$_3$ in 1.4 mL of water, as depicted in Table 2. Then, the solution was purged with nitrogen for 5 minutes for oxygen displacement. Finally, the solution was placed in polypropylene cylindrical containers with a diameter of 8 mm in a water bath at (37±1) $^\circ$C for 24 hours. The reactions were designed with an excess of HEMA so that all the boron was included in the material. Thus, for the higher boric acid concentration used, the HEMA concentration was two times higher.

**Table 2:** Synthesis reaction conditions of poly(HEMA)$^{10}$B.

H$_2$O (mL)	Boric acid (mg)	HEMA (mL)	APS (mg)	TEMED (mL)
1.4	$0.00\times 10^0$	0.4	20.0	0.2
1.4	$1.00\times 10^0$	0.4	20.0	0.2
1.4	$3.00\times 10^0$	0.4	20.0	0.2
1.4	$1.00\times 10^1$	0.4	20.0	0.2
1.4	$2.00\times 10^1$	0.4	20.0	0.2
1.4	$3.00\times 10^1$	0.4	20.0	0.2
1.4	$6.00\times 10^1$	0.4	20.0	0.2
1.4	$1.00\times 10^2$	0.4	20.0	0.2

### Synthesis yield estimation method

The synthesis yields in the poly(HEMA)$^{10}$B syntheses with different concentrations of boric acid was defined as the amount of HEMA included in the polymeric material over the total amount of HEMA in the synthesis. Therefore, the unreacted HEMA was quantified by means of the Jones oxidation of the alcohol groups in the molecule^61^. For that purpose, Jones reagent was prepared by dissolving 2.67 g of Cr$_2$O$_3$ (99.9 % Sigma-Aldrich, USA) in 2.3 mL of H$_2$SO$_4$ (99.999 % Merk) in an ice bath for at least 1 h. Afterwards, 10 mL of Milli-Q grade water was added drop by drop and the obtained solution was left at ambient temperature for its stabilization. The oxidation reaction was carried out by adding 0.4 mL of the remanent liquid phase of the reaction product to 1.1 mL of water and 1 mL of the Jones reagent, followed by gentle mixing and for a reaction time of 30 min. Then, 0.4 mL of the obtained solution was diluted 10 times with water. The final solution was analyzed by means of UV-Vis spectroscopy using a Shimadzu UV-1800 spectrophotometer (Japan) between 400 and 800 nm, and the absorbance at 589 nm was used for the quantification. A calibration curve was measured with known quantities of HEMA (0.000206, 0.000412, 0.000824, 0.001240, 0.001650 moles) in a 1.5 mL volume of water; then 1 mL of the Jones reagent was added and the oxidation reaction was carried out for 30 minutes. The used calibration curve is shown in Supplementary Fig. S1 online.

### $^1$H NMR spectroscopy

The molecular interactions in the obtained material were confirmed by proton nuclear magnetic resonance ($^1$H NMR) spectroscopy determining the interaction between boric acid and HEMA in the polymer network sustained by boron cross-linking. $^1$H NMR studies were carried out in a NMR Bruker Avance 400 MHz spectrometer, with samples containing 3.6 $\upmu$L of HEMA, 1.8 mg of H$_3$ $^{10}$BO$_3$ or a mixture of both compounds in 1 mL of D$_2$H.

### Boron release kinetics

The poly(HEMA)$^{10}$B was dried at 50 $^\circ$C until constant weight, then ground with liquid air and separated with a 100-mesh sieve. The obtained particles with a mean size below 149 $\upmu$m were suspended in 1 mL simulated body fluid (SBF) solution and further poured through a syringe with a 21-G needle (inner diameter of 514 $\upmu$m). The SBF solution was prepared according to Marques et al.^47^ using all reagents supplied by Sigma-Aldrich, USA without any further processing. A total amount of 100 mg of dried particles of the synthesized material was washed with 5 mL of distilled water to remove any remaining monomer in the material, and then suspended in the SBF solution for different periods of time. For that purpose, 40 mL samples of SBF were incubated in a thermal bath (Haake Technik GmbH, Germany) at (37±0.5) $^\circ$C for 10, 30, 60, 240 and 1440 min. After the corresponding incubation time, the solution was vacuum filtered, washed with 60 mL of distilled water and dried in an air oven at (50±0.5) $^\circ$C until constant weight. Finally, poly(HEMA)$^{10}$B released content was estimated gravimetrically. The obtained results were fitted according to a pseudo-first kinetics model representing the polymer hydrolysis as the main degradation mechanism^48^.

### X-ray irradiations

For the X-ray irradiations, a conventional X-ray tube coupled to a W anode with a maximum power of 3 kW and a Kristalloflex (Siemens, Germany) generator operating with 44 kVp and 44 mA was used. Samples were placed at 830 mm from the source and a 46 $\times$ 58 mm$^2$ collimator was used at 782 mm from the source. With this configuration, a dose rate of (790 ± 20) mGy/min was determined by a calibrated Farmer-type ionization chamber (TN 30013, PTW, Freiburg, Germany) with the corresponding temperature and pressure corrections. Samples were irradiated for the required time to deliver dose values from 0.4 to 7.2 Gy.

### Neutron-gamma irradiations

The RA-0 Nuclear Reactor, a zero power reactor that belongs to the National Atomic Energy Commission (CNEA) and it is currently placed at the Universidad Nacional de Córdoba (UNC), Argentina, was used as a neutron source^40^. The reactor normally operates at a power of 1 W. The fuel elements are vertically located in an aluminum ring structure and are composed of a mixture of UO$_2$ enriched 20 % in $^{235}$U, graphite and pitch located inside an aluminum sheath with an active length of 54 cm, while light water is used as a neutron moderator. The samples were placed in the central irradiation channel of the reactor core. The dose absorbed by each dosimeter was calculated by means of the Monte Carlo transport code MCNP according to the procedure described elsewhere^40^. In the simulations, the contributions from photons, neutron elastic scattering and neutron absorption were discriminated so that the capture events could be quantified.

### MCNP simulations

The MCNP dose calculations were made using version 6.1 of the code^62^ in a PC with an Intel Core i7-3930K CPU 3.20 GHz x12 processor with 32 Gb RAM, by using the KCODE card with 5 $\times$ 10$^5$ source histories per cycle and 500 active cycles. The used tallies were the flux average over a cell (F4n), the energy deposition (F6n) and the FM card with special MCNP reaction numbers to discriminate between the different dose contributions. Samples were irradiated in the RA-0 nuclear reactor at different doses accounting the thermal neutron capture in $^{10}$B interaction, according to the calculations by Monte Carlo simulations shown in the “Thermal neutron dose calculation” section of the supplementary information.

### Morphological characterization

Morphological studies of the irradiated samples were performed by scanning electron microscopy (SEM) using a SEM Carl Zeiss Sigma microscope. Samples of about 10 mm$^3$ were freeze dried in a L-T8 lyophilizer (Rificor, Argentina) at a pressure of 0.01 mbar and a temperature of (− 48.0 ± 0.1) $^\circ$C for 8 h. Subsequently, samples were fractured to expose the inner structure of the material and were splatter-coated with gold and scanned at 5.00 kV with a magnification ranging from 40X to 71000X. Three samples were selected for this study, a non-irradiated sample, an X-ray only irradiated sample at a dose of 5.50 Gy and a sample irradiated with a neutron-gamma field with a gamma dose of (5.50 $+$ 0.77) Gy and a dose of 18.01 Gy due to fragments released during the neutron capture in $^{10}$B isotope.

### Rheology

The mechanical properties of the poly(HEMA)$^{10}$B samples were studied by oscillatory rheology in an Anton Paar MCR 301 rheometer with an 8 mm diameter parallel plate geometry (PP08). To carry out the measurements, samples were cut into discs of 1 to 2 mm thickness and 8 mm diameter. A strain sweep test was performed on each sample between 0.1 and 100 % at a 1 Hz frequency to determine the linear viscoelastic range (LVR). Subsequently, the elastic modulus of each sample was studied using a frequency sweep between 0.1 and 100 Hz at the previously determined LVR, recording five points per decade by triplicate at (20.0 ± 0.1) $^\circ$C. The values of the elastic modulus at 10 Hz were used since for these frequency the behavior of the material as a solid prevails. Additionally, in order to reduce dispersion in the analytic technique due to synthesis yield differences in the poly(HEMA)$^{10}$B synthesis, the elastic modulus values were normalized by each sample synthesis yield value.

For analyzing the response of the material exposed to different radiation doses, the values of the measured elastic modulus at 10 Hz were normalized by each sample synthesis yield value in order to reduce the dispersion caused by differences in the poly(HEMA)$^{10}$B synthesis. The reported error bars were computed considering four independent uncertainty sources; (a) the rheometry readout technique itself, contributing with a 1.81 % according to the Anton Paar MCR 301 rheometer technical specifications; (b) the uncertainty of the synthesis yield estimation method, contributing with a 1.02 % mainly due to the glassware used for preparing the solutions; (c) the uncertainty in the X-ray irradiations, contributing with a 1.14 %; and (d) the neutron dose estimation method, based on the MCNP simulations and taking into account the correction for the power rise of the nuclear reactor, as detailed in the Supplementary information, contributing with a 2.46 % to the overall uncertainty. Thus, items (a) to (c) determine an uncertainty of 3.97 % for the response of the polymer to X-rays and items (a) to (d) determine an uncertainty of 6.43 % when analysing the response to thermal neutron dose.

### Affected volume estimation

Ionizing radiations induce changes in the mechanical properties of the poly(HEMA)$^{10}$B samples. In fact, poly(HEMA)$^{10}$B samples irradiated with neutrons undergo nuclear reactions, like neutron capture in $^{10}$B, thus nuclear fragments and prompt gamma are produced as residuals. Effects on the poly(HEMA)$^{10}$B mechanical properties due to indirect ionizing radiations may not be significant, but high linear energy transfer (LET) projectiles like $^7$Li and $^4$He, or alpha particle, may actually produce non-negligible effects on the polymeric network structure.

The proposed approach consists on assessing the volume that may be affected by the high LET radiations. Thereby, FLUKA simulations were carried out considering 0.84 MeV $^7$Li and 1.47 MeV $^4$He projectiles emitted in the positions of the neutron capture reactions, previously determined by means of MCNP simulations. Spatial distributions of those high LET particles, deposited energy and charge produced were obtained by FLUKA simulations, accounting for particle range and lateral extension of the deposited energy or charge produced around the track. The volume directly affected for each particle was approximated to a cone, with height and base corresponding to the range and lateral extension, respectively. Hence, the net affected volume was obtained by adding the volumes of such cones.

### FLUKA simulations

The FLUKA Monte Carlo main code^63^ is suitable for heavy ions simulations^64^, therefore it was used to simulate the transport of $^7$Li and $^4$He ions in poly(HEMA)$^{10}$B. Chemical and corresponding isotopic compositions were used to define the physical properties of poly(HEMA)$^{10}$B in FLUKA. A mass density of (1.101 ± 0.033) g/mL was experimentally determined for a concentration of 5 % w/v of boric acid. This value, along with sample dimensions were introduced in the material definition in the simulations. The HADROTHE defaults with active ion transport were used along with default values for absorption energy cut-off. Calculations were performed in the i7-processor PC computing 1$\times$10$^8$ primary showers in all cases.

### Tensile tests

A tensile test was performed according to the norm ASTM D 638-10 (Standard Test Method for Tensile Properties of Plastics) using a 23-5S Instron-Emic mechanical analyzer (Brazil), with a 50 N load cell. Samples were cut with average dimensions of (8.0 ± 0.3) mm length, (2.0 ± 0.3) mm thickness and (1.8 ± 0.3) mm width. Samples were dried at 37 $^\circ$C until a constant mass was obtained. Subsequently, all samples with a mass of about (27 ± 9) mg were rehydrated by adding a volume of distilled water equivalent to the average water content of all samples, previously estimated as 45 %wt. Samples were rehydrated for 5 days before the tensile test. Afterwards, they were placed in jaws with an initial gap of (3.00 ± 0.01) mm and displacement speed of 2.00 mm/min. All tests were carried out at 25 $^\circ$C.

For analyzing the response of the material exposed to different radiation doses, the values of the elongation at break $\Delta$L/L$_0$ were plotted, by considering the ratio of the elongation variation $\Delta$L over the initial longitude L$_0$. In an analogue way to the uncertainty analysis presented for rheology, three independent uncertainty sources were considered; (a) the tensile stress technique itself, contributing with a 1.00 % according to the ASTM norm; (b) the uncertainty in the X-ray irradiations, contributing with a 1.14 %; and (c) the neutron dose estimation method, contributing with a 2.46 % to the overall uncertainty. Thus, items (a) to (c) determine an uncertainty of 4.60 % for the response of the polymer when analysed by the tensile technique.

## Supplementary Information

Supplementary Video 1.

Supplementary Legend.

Supplementary Information.

## Data Availability

Access to Monte Carlo main codes MCNP6 and FLUKA was available by official license (NEA and Los Alamos Laboratory) addressed to Prof. Mauro Valente, NEA liaison officer for Argentina-South America region.
