# Supplementary material for: Smart material based on boron crosslinked polymers with potential applications in cancer radiation therapy
Source: Sci Rep. 2021 Jun 10;11:12269. doi: 10.1038/s41598-021-91413-x (PMC8192942; doi:10.1038/s41598-021-91413-x)
Supplement: Supplementary file 2 — Supplementary Legend. [file 41598_2021_91413_MOESM2_ESM.docx]

**Supplementary Video**

Title: Flow test

Legend: Dehydrated and micronized poly(HEMA)^10^B suspended in SBF solution flowing through a standard 21-G needle.
