# Supplementary material for: Smart material based on boron crosslinked polymers with potential applications in cancer radiation therapy
Source: Sci Rep. 2021 Jun 10;11:12269. doi: 10.1038/s41598-021-91413-x (PMC8192942; doi:10.1038/s41598-021-91413-x)
Supplement: Supplementary file 3 — Supplementary Information. [file 41598_2021_91413_MOESM3_ESM.pdf]

Supplementary information

Smart material based on boron crosslinked polymers with potential applications in cancer radiation therapy

José Vedelago[1,2,+,a], Facundo Mattea[3,4,2], Sebastían Triviño[5,6,2], María del Mar Montesinos[7], Walter Keil[6], Mauro Valente[1,2,8,*], Marcelo Romero[3,4,2,+,**]

[1]Instituto de Física Enrique Gaviola (IFEG), CONICET, Córdoba, X5000HUA, Argentina.
[2]Laboratorio de Investigación e Instrumentación en Física Aplicada a la Medicina e Imágenes por Rayos X (LIIFAMIRx), FAMAF-UNC, Córdoba, X5000HUA, Argentina.
[3]Departamento de Química Orgánica, FCQ-UNC, Córdoba, X5000HUA, Argentina.
[4]Instituto de Investigación y Desarrollo en Ingeniería de Procesos y Química Aplicada (IPQA), CONICET, Córdoba, X5000HUA, Argentina.
[5]Centro de Medicina Nuclear y Radioterapia Patagonia Austral, Río Gallegos, Z9400, Argentina.
[6]UNC - FCEFyN & CNEA - Reactor Nuclear RA-0, Córdoba, X5000HUA, Argentina.
[7]Centro de Investigaciones en Bioquímica Clínica e Inmunología (CIBICI), CONICET and Departamento de Bioquímica Clínica, Facultad de Ciencias Químicas, Universidad Nacional de Córdoba, X5000HUA, Argentina.
[8]Centro de Física e Ingeniería en Medicina (CFIM) & Departamento de Ciencias Físicas, Universidad de La Frontera, Temuco, Casilla 54-D, Chile.
[a]Current affiliation: Division of Medical Physics in Radiation Oncology, German Cancer Research Center (DKFZ), 69120 Heidelberg, Germany.
*mauro.valente@gmail.com
**marceloricardoromero@gmail.com
[+]these authors contributed equally to this work

Submitted to *Scientific Reports* in October 2019 (revised version January 2021).

Synthesis yields estimation in the poly(HEMA)^{10}B synthesis

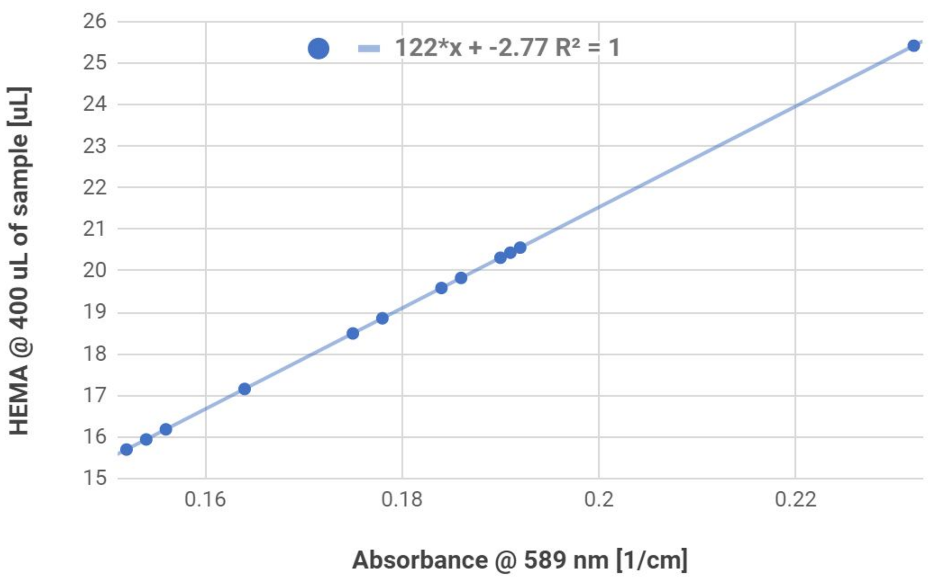

Supplementary Figure S1: Calibration curve used for the synthesis yields estimation in the poly(HEMA)^{10}B synthesis.

Fitting parameters corresponding to Fig. 6: Quadratic fit

Model Poly2:
 f(x) = p1*x^2 + p2*x + p3
Coefficients (with 95% confidence bounds):
 p1 = -0.003147 (-0.01189, 0.005592)
 p2 = 0.0442 (-0.01894, 0.1073)
 p3 = 0.9821 (0.9, 1.064)
Goodness of fit:
  SSE: 0.01107
  R-square: 0.6939
  Adjusted R-square: 0.5714
  RMSE: 0.04705
χ^2 = 1.6187

Fitting parameters corresponding to Fig. 8: Linear fit

Model Poly1:
 f(x) = p1*x + p2
Coefficients (with 95% confidence bounds):
 p1 = -0.008707 (-0.01382, -0.003597)
 p2 = 0.9404 (0.857, 1.024)
Goodness of fit:
  SSE: 0.02167
  R-square: 0.6587
  Adjusted R-square: 0.616
  RMSE: 0.05204
χ^2 = 0.7214

Thermal neutron dose calculation

The thermal neutron dose for the RA-0 nuclear reactor operating at 1 W was calculated from the MCNP simulations according to the dose rates values depicted in Supplementary Table S1. The radiation received by the samples during the set-up of the RA-0 nuclear reactor was approximated by fitting the exponential power rise depicted in Supplementary Fig. S2.

RA-0 central reflector position [mm]	Mean thermal neutron dose rate [Gy/min]	Mean thermal neutron dose rate error [Gy/min]
140	0.2908	0.0004
70	0.3258	0.0003
0	0.3567	0.0003
-70	0.3383	0.0003

Supplementary Table S1: Mean thermal neutron dose rate calculated by Monte Carlo simulations.

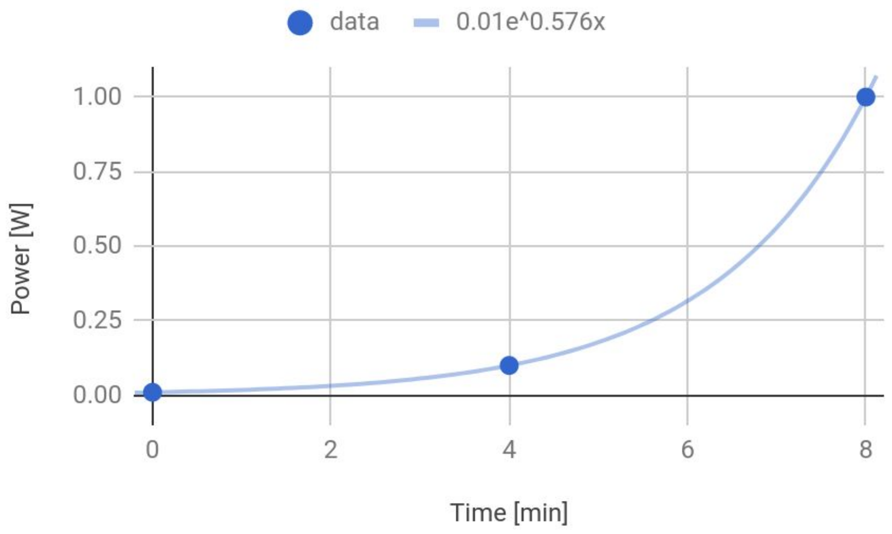

Supplementary Figure S2: Power-rise of the RA-0 nuclear reactor.

Thus, the following integral can be computed:

$$I = \int_{0}^{8\,min} a\ \exp(tb)\,dt\ \simeq 1.72\ W/min$$

where $a = 0.01\ W$ and $b = 0.576\ min^{-1}$.

Therefore, in each irradiation, an equivalent to exposure to 1 W during 1.72 min was added. Irradiations were carried out in four reactor operations of 12; 24; 24 and 36 minutes at a power of 1 W. The resulting dose contribution to the total absorbed dose due to the neutron capture reactions, calculated by the Monte Carlo simulation and corrected by the time elapsed for reaching the desired power, are depicted in Supplementary Fig. S3. The ‘Samples ID’ are the labels assigned to each sample.

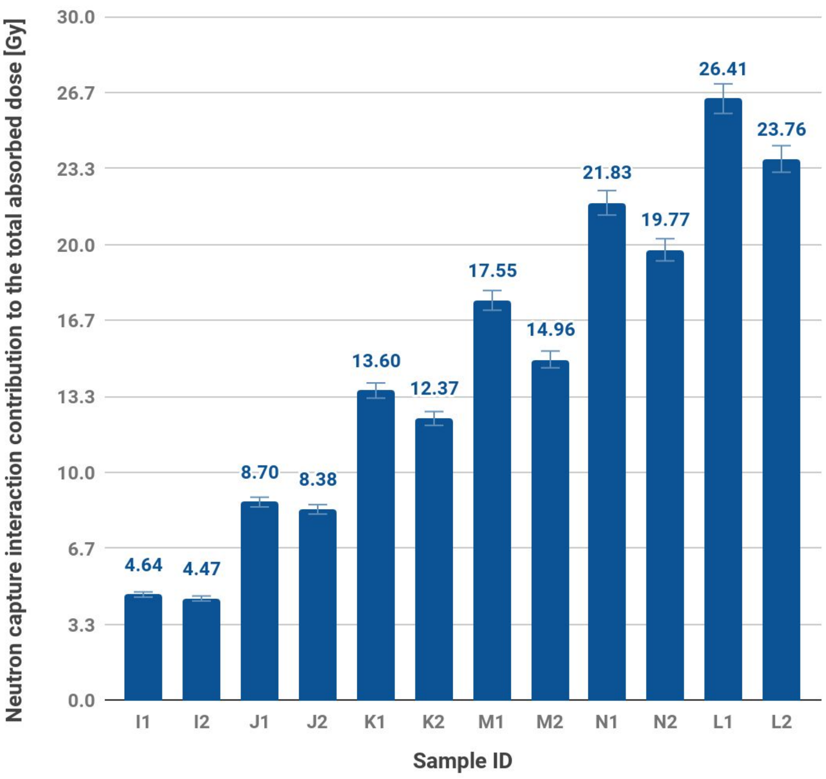

Supplementary Figure S3: Thermal neutron dose absorbed for each poly(HEMA)^{10}B sample.
